# Climate Change: A Review of Its Health Impact and Percieved Awareness by the Young Citizens

**DOI:** 10.5539/gjhs.v6n4p196

**Published:** 2014-04-16

**Authors:** Muhammad Sabbir Rahman, Osman Bin Mohamad, Zainal bin Abu Zarim

**Affiliations:** 1 Faculty of Languages and Management, International Islamic University Malaysia, Kuala Lumpur, Malaysia; 2 Graduate School of Management, Multimedia University, Melaka, Malaysia

**Keywords:** climate change, physical impact, psychological impact, young citizens, Malaysia

## Abstract

In recent time climate change and its impact on human health and awareness constitute a set of complex and serious consequences to be tackled by an individual country. Climate change is not merely an environmental issue, but also it is a threat that goes beyond national borders. The purpose of this study is to identify the awareness and the impact of climate change, perceived by the young citizens in Malaysia by focusing on gender differences. Based on a survey of 200 respondents from different public and private University’s students in Malaysia, this research used descriptive statistics and T-test to look into the research objective. The results revealed media can play an important role in the awareness of climate change. Meanwhile the male respondents have shown considerable attention on the physical impact of climate change like heat related stress. On the other hand female respondents have shown considerable attention to the psychological impact by the climate change. From a pragmatic perspective, the findings from this research will assists the policy makers to understand more about the perceived awareness on the climate change issues of the young citizens which ultimately assist them to inaugurate new initiatives to confront the challenges of climate changes. This research is among the pioneer study on the issue of the perceived awareness in regards to climate change in Malaysia by focusing on gender differences.

## 1. Introduction

In recent time climate change is viewed as one of the most significant global problems which affect directly or indirectly alter the structure of the environment (United Nations Framework Convention on Climate Change, 1992; Costello et al., 2009). The impact of climate change on the environment and human health is far reaching and thus influencing social changes in many aspects. The impact of climate change has seen in numerous aspects, for instance human health, ecosystem health and biodiversity, food production, economic growth, tourism, and water resources (Kovats et al., 2005; Ebi et al., 2006; Arnell, 2004).

Because of climate change the world is now experiencing warming effects with increasing atmospheric greenhouse gases from intensive use of fossil fuel. According to a study by IPCC (2007) the surface temperatures of global have risen per decade average by 0.13°C since 1950. At the same time global average surface temperatures can be increased from 1.8°C to 4°C by the end of the 21st century subject on the extent of future GHG emissions. In addition to this the climate will continue to warm in the next few decades (IPCC, 2007).

In fact, Geographically Malaysia is located at western part of the Maritime Continent between the pacific ocean to the east and Indian ocean to the west. Hence the climate of Malaysia is naturally influenced by the Southeast Asia Maritime Continent monsoon and climate changes variability accompanying with this two large oceans. There are two regional monsoon system, namely southwest monsoon accompanied by southwesterly winds (May to August) and northeast monsoon dominated by northeasterly winds (November to February). During southwest wind strong pulses of cold surge, wind penetrates to the most southern region of the South China Sea and the region is typically wetter in the northeast monsoon since the Inter-tropical Convergence Zone (ITCZ) is closely located to the equator.

In northwestward period the incidents of significant and prolonged forest fires in Sumatra and Kalimantan cause severe haze in Malaysia and affect the climate in Malaysia. In recent years there has been a significant increase in the intensity of rainfall over Peninsular Malaysia during the northeast monsoon causing major floods in Malaysia (Chang et al., 2005). Due to the geographic setting it is easy for pollution to spread up in Malaysia and therefore, causes changes in climate and impact Malaysia’s subtle environmental equilibrium (Tangang & Juneng, 2011).

In the Malay majority of research in regards to climate change issues, mainly looked at the adaptation perspective by putting huge importance on improving ecosystem management, management of water resource, and sustainable agricultural productive, effective and efficient resource utilisation by maximising economic benefits for the motherland (Sloar, 2011).

Solar (2011) also mentioned that till date Malaysian government has able to initiate substantial mount of activities through various programs to confront the challenges of climate change. According to Pereira and Subramaniam (2007) climate change in Malaysia involving more than environmental issues, for instance economic growth and human welfare. In addition, in Malaysia youth are prospective constituents in affecting future policies of the nation. The population of Malaysia is approximately 27.3 million, which is growing at a rate of 2%, the proportion of young population (27.2%) under 30 years are expected to equal more than half of the total population in 2015 (Zarinah, 2011). Perceived awareness of young citizens in regards to climate change is critical as for social context within which policy makers operate. That is why this research, particularly focuses on Malaysian young citizens perceived awareness of health related issues due to the impact of the climate change.

On the other hand under western country’s perspective the wealth of literature concerning general perceptions of climate change and awareness of the outcome is enormous. In developed countries the level of awareness on the issues of climate changes is very high. For instance, only 1% of the English have not heard of either ‘climate change’, ‘global warming’ or the ‘greenhouse effect’ (DEFRA, 2002; Lorenzon et al., 2007). In addition, 92% of Americans are aware of global warming by 76% have already viewed climate change caused a serious problem in all aspects of human life (Leiserowitz, 2003; PIPA, 2005). However, the context of perceived awareness on the issues like environment, controlling energy consumption and health related by the young segments takes a low priority by previous academic researchers (Poortinga & Pidgeon, 2003; DEFRA, 2002; Norton & Leaman, 2004; Bostrom et al., 1994; Kempton, 1997; Bord et al., 1998; Poortinga et al., 2006; Leiserowitz, 2006).

In further this research will be a sound direction for the policy makers for prevention measures with including this important segment. As a whole this study tries to explore Malaysian young citizens’ perceived awareness about the impact of climate change. The overall objective of this research is to discover the young citizens perceived awareness of climate change and its impact on the human health by focusing on gender differences. In line with that this research answer following research questions to answer such as:

- What is the level of perceived awareness of the physical and psychological impact of the climate change?
- Does this level of perceived awareness differ based on gender?

## 2. Literature Review

Climate change consists of atmospheric temperature rise, melting of glaciers, occurrence of disease, sinking islands, sea-level rising, water scarcity, increased mental stress and disease, augmented illness from diarrhoeal disease and endemic mortality, rising of coastal water temperature associated with aggravate toxicity of cholera (Halady and Rao, 2009; Fischoff, 1995). Every nation in the world in one way to another contributed the phenomena to the climate change which consists of industrialization, burning fossil fuels, deforestation, and unlimited use of resources and vigor contributing to GHG emissions (ADB, 2008). Due to climate change common transmissible diseases subtle and endemic to climate change are malaria and cholera, meningococcal meningitis, dengue, leptospirosis and rickettsial infections.

### 2.1 Climate Change and its impact on Human Health

#### 2.1.1 Physical Effects

Climate change sensitive diseases comprise of heat-related diseases, waterborne diseases, vector borne diseases, diseases cause from air pollution and diseases related to extreme weather conditions such as floods, droughts, windstorms and fires. Mostly the water borne ailments are spread by the flood and induces pollution extremely when rainwater floods the urban metropolis. The flood water overflown in the urban area is highly polluted and leads to considerable pollution in the natural water sources (Hasan & Mulamoottil, 1994). For example, such devastating events occurred in southern Malaysia from mid-December 2006 until late January 2007 where approximate 200,000 citizens were affected by the flood along with 16 testified deaths (Chang et al., 2005; Tangang, 2011).

Due to climate change, increased difficulties in access to quality water sources contribute to the disease burden which ultimately hampering the freedom to live a long and healthy life. As the rainfall pattern is being changed as a result the supply of quantity and quality of water also become scarce. As consequences warmer condition of climate is adversely affecting the potential levels of aquatic-borne Pathogens disease specially cholera, malaria and dengue (Shiklomanov, 2001; Rodo et al., 2002; Agrawala et al., 2003). Additionally, in Malaysia’s government as well as the citizens face a significant challenge to control of vector-borne and food-and-water-borne diarrheal diseases (Husaini, 2007).

Worldwide warmer temperatures contributing of breeding of larger mosquito and other insect which potentially carry malaria, unclean water from flooding increases the incidences of cholera (Halady & Rao, 2009). The environment is undergoing higher levels of contamination associated with warmer surroundings which is leading to a greater risk of mortality rate resulting from respiratory Diseases (WHO, 2001). Therefore, generally warmer climate and its simultaneous effects of high temperature on atmospheric pollution lead to greater mortality overall. According to Roda et al. (2002) climate change contributed approximately 5.5 million deaths of human lives in 2000. Among them 2.8 million were passed due to malnutrition; another 1.5 million was due to diarrhoea, malaria contributed to another 1 million live. In addition, another 200 thousand was lost because of flood and surprisingly, nearly half of all worldwide was lost in East and South Asia region.

#### 2.1.2 Psychological Effects

Physiological effects on human body due to temperature are well-known, since extreme conditions of heat or cold may damage to many body functions. In fact, researchers have found out some negative emotional reactions in certain individuals due to climate change (Fritze et al., 2008). Medical practitioners have also experienced with the increased amount of patients with anxiety, depression (Miller, 2008). Negative psychological circumstances relating to climate change are frequently appearing in public opinion, comentatory polls as well as in the psychological literature (Searle & Gow, 2009; Fritze et al., 2008). While some individuals are having intense worry that leads to distress and/or interferes with everyday life (Fritze et al., 2008). Research has exhibited that trait anxiety and future anxiety both are concomitant with an interpretive bias for future circumstances (Zaleski, 1996; Rapee, 1997; Eysenck & Derakshan, 1997; Butler & Mathews, 1987; Goldstein et al., 2002).

Numerous researchers have found a strong relationship between climate change and stress (Swim et al., 2009; Gifford, 2008; Kazdin, 2009). In addition the researchers also observe that the consensus of the psychologist has grown tremendously on the impact of environmental problems and its effect on their behavioural aspects (Gardner & Stern, 2002; Geller, 2002; Gifford, 2007; Oskamp & Schultz, 2006; Swim et al., 2009; Vlek & Steg, 2007). However the report from IPPC (2007) identified that human behaviour and its relationship with climate change components has taken least consideration. In fact, there does seem to be emerging evidence that generally people are concerned regarding environmental problems and its impact on their physiological aspect (Gow & Leahy, 2005).

## 3. Methodology

The population for this research comprised of students under various private and public Universities in Malaysia. The choice of respondents included in the survey by using a structured questionnaire. Data were collected through the university campus intercept procedure. The main part of the questionnaire was divided into three parts, for instance perceived awareness on climate change, physical impact and psychological impact. The questionnaire was three pages long, including a cover a letter on the first page and necessary demographic question on the last page. Participants were also asked their age, gender, mode of study and their awareness about physical and psychological impact by the climate change. All the details of the questionnaires were operationalised by adapting from the research of Solar (2011) and Menny et al. (2013) which were measured a seven point Likert scale response format such that: 1= Strongly disagree to 7= Strongly agree (Lin & Lee, 2004).

The items of the instrument were adapted from various scholars research instrument. For instance, awareness on climate change was measured by 10 item scale adapted from the Solar (2011) and Menny et al. (2013). Physical impact was operationalised using 3 items adapted from Solar (2011) and Menny et al. (2013). In measuring psychological impact, we adapted from Solar (2011) -2items and Menny et al. (2013)-1item measures. This research established content validity through pre examining the instruments with 10 respondents with five males (MBA students) and five females (MBA students) from one private university. An internal testing process was taken out with the suggestion from academics and scholars. After adjusting the input from the pretesting stage a total of 250 questionnaires were distributed using face to face survey procedure. As this research applied convenient sampling procedure for data collection from the active students’ under various private and public universities in Malaysia by focusing gender differences. This research used convenience sampling as participants in this research were readily available and agreed to participate in this research (Fink, 1995; Frey et al., 2000; Henry, 1990). As a convenience sampling includes only respondents ready and available, there is no excuse for sloppiness (Babbie, 1990). According to Babbie (1990) to present the population researcher can use convenience sampling to achieve their research objective. The survey questionnaires were distributed to the targeted respondents and collected between the September 2012 and November 2012. In aggregate, 50 surveys were eliminated as unusable due to incomplete data. The final sample (n=200) was included in data analysis; 120 cases of male and 80 from female respondents responded. The reliability of this scale was assessed through the internal consistency measurement by using Cronbach’s coefficient alpha. The result from reliability on the basis of Cronbach’s alpha shows that the criteria for individual contract exceed Nunnally and Bernstein’s (1994) standard of 0.70. Awareness on climate change was measured by 9 item scale with alpha=0. 826. Physical impact was operationalised using 3items which also proved adequately reliable (alpha=0.821). Selected scales of psychological impact had good reliability (alpha=0. 812).

Aside from a descriptive survey of the demographic profile, this research also applied independently-samples t-tests to compare the average scores of two different groups (male and female). The researchers believe that the operationalisation of independent-samples t-test explores whether there is a statistically significant difference in the mean scores for the two groups (i.e. Whether males and females differ significantly in terms of their awareness on climate change in regards of physical and psychological impact).

## 4. Results Discussion

Out of 200 male respondents (60%) made up the majority followed by female (40%). Most of the respondents were Malays (60%), followed by Chinese (35%) and Indian (5%). The majority of the respondent’s response came from students of private universities (70%) followed by public universities (30%). Most of the students were from undergraduate level (60%), followed by postgraduate (40%). In terms of their general belief about the climate change 70% of the respondents believed that the climate of their motherland has changed significantly in the last 10years. The interestingly majority of the respondents (80%) agreed that the issue of climate change and its discussion belonging all to scientific in nature. Even though a significant number of respondents (70%) think that climate change has several impacts on their personal and social function.

In fact, all of the respondents agreed that they observed, there is a steady rise in the average temperature of their current living situation. Most of the respondents (90%) agreed that over the year their respective institution co-curricular activity centre have taken a considerable program for the mitigation of contamination of the environment. Ultimately, the majority of the respondents perceived that every member of the society, including the governments are affected by the climate change and need to act proactive steps in this matter. Apart from that majority of the respondents (90%) were concerned about the availability and accessibility of information on the phenomenon through the mass media (radio/TV, internet, social media and newspaper). This research also explored that majority of the respondents (78%) believed that the university’s authorities can play an important role with aware their students by highlighting the major impact of climate change.

The respondents were asked to answer about their current awareness on the climate change and the role of media to enhance the awareness level of climate change. Table 1 shows the result of preferred sources to enhance the awareness level of climate change perceived by the respondents. The results dictate that for female respondents awareness through community engagement is the most preferred way to raise the awareness level of climate change, while environmental pollution causes the extreme storms, heat, heavy rain and flooding are among the more concerned by the male respondents. Although both groups of respondents (Male and Female) show significantly similar importance on the part of the media as one of the important antecedents to enhance the awareness of climate change among the citizens in Malaysia. However, female respondents score significantly higher than male respondents in regards of overall awareness and consequences of climate change among the young citizens studying in different universities in Malaysia. In regards to the possible impact by the climate change both physical, environmental and psychological aspects female respondents indicate a significantly higher score compared to male respondents. It is within expectation to see that both groups of respondents are significantly agreed that they are afraid of the possible consequences of the climate change in future.

**Table 1:** Perceived awareness on climate change

Items	MALE		FEMALE		Comparison	

	Mean	SD	Mean	SD	*t-*statistics	*p-*value
Awareness of the effects of climate change through community involvement	4.91	1.215	5.25	1.077	-2.121	.035*
Community involvement in mitigating the environmental pollution	5.01	1.041	5.29	.921	-2.004	.046*
Improved climate impact by integrating water resources management system (approaches to mitigate the climate change)	5.07	1.069	5.24	.919	-1.191	.235
Media role in informing about the topic of climate change	5.39	1.092	5.42	.992	-1.528	.012**
Awareness of Environmental damages	5.66	1.050	5.32	.961	-1.955	.032*
The issue of climate change must take an important consideration by the respective groups (researchers, policy makers)	5.71	1.142	5.86	.897	-1.010	.309
Young citizens studying in Malaysia understand the effects of climate change	5.51	1.196	5.77	.960	-1.055	.028**
Mass media can play an important role to aware the climate change	5.67	1.002	5.76	.970	-.666	.050
Are you aware about the negative impact of the potential effects of climate change	5.79	1.012	5.81	.960	-0.416	.001**

Notes: Significant at:

* 0.05 and

** 0.01 levels.

As shown in Table 2, respondents from both groups demonstrate similar patterns of the issues regarding climate change and its impact on malaria and being afraid of the potential physical consequences of the effect of climate change. Surprisingly, male respondents demonstrate significantly higher perceived concerns about the impact of climate change on the temperature increase during summer time which causes heat related stress among the citizens. Table 3 shows the possible perceived consequence of the climate change on the individual psychological impact. It is interesting to note that female respondents have shown a significant difference with male respondents in terms of individual stress, anxiety and depression due to climate change impact.

**Table 2:** Perceived physical impact of climate change

Items	MALE		FEMALE		Comparison	
	Mean	SD	Mean	SD	t-statistics	p-value
Climate change has an impact on malaria	5.59	1.208	5.64	.954	-1.687	.003**
Climate change has an impact on the increase in summer temperature – increase in heat related stress	5.31	.998	5.24	1.093	.486	.042*
Are you afraid of the possible physical consequences from the consequence of climate change	5.30	1.051	5.38	1.109	-1.832	-.280

Notes: Significant at:

* 0.05 and

** 0.01 levels.

**Table 3:** Perceived psychological impact of climate change

Items	MALE		FEMALE		Comparison	
	Mean	SD	Mean	SD	t-statistics	p-value
Climate change has an impact on stress	5.23	1.147	5.48	1.005	-1.653	.010**
Climate change has an impact on anxiety	5.34	1.113	5.68	.968	-2.303	.002**
Climate change has an impact on depression	5.47	1.114	5.70	.934	-1.597	.020*

Notes: Significant at:

* 0.05 and

** 0.01 levels.

## 5. Discussion and Implication

This research has contributed to the climate change research in various aspects. Most importantly, it has bridged the gap on the scarcity of research in investigating the perceived behavioural understanding by the younger citizens in Malaysia towards climate change and its possible impact on physical and psychological aspects by focusing on the gender differences. The role of T-test by looking for the comparative study in between male and female, as this research also enters into deeper on the issue on the impact of climate changes. One major contribution made by this research is that there are significant differences and similarities exist in the awareness of climate changes, physical and psychological impact of the climate changes issues in between male and female respondents in Malaysia which were not taken a research consideration by the past academic researchers.

Apart from that the findings from the statistical results reveal that both groups of respondents expressed strong agreement on the role of media in the awareness of climate change (Fritze et al., 2008). On the other hand female respondents were more interested in developing the awareness of climate changes through mass community participation activities. Similarly, both groups of respondents significantly agreed that they are afraid of the possible impact by the climate changes (Searle & Gow, 2009; Fritze et al., 2008). The results also indicate that both groups of respondents are quite aware about the potential physical and psychological impact of the climate changes (Swim et al., 2009; Gifford, 2008; Kazdin, 2009). Even though female respondents had shown significantly higher concern on the climate change and its impact on the individual anxiety. The result also suggests that male respondents had shown a significant amount of concern on the increase of temperature and its possible impact on the heat related stress among the citizens.

The findings from this research validate the contention that there is indeed significant concern have arisen in the judgment of the young citizens in Malaysia about the issue of climate change and its possible impact on physical and psychological aspects. In that regards the respective department from the Universities and Government must take initiatives to aware more about climate change in all levels of students. The government and respective agency both local and private need to circulate accurate and reliable information about the climate change among the member of a particular society to create the initiative to face the challenges of climate changes. In fact, to mitigate the problem of climate change young citizens obviously play an important role to aware about the possible impact of climate change towards the mass people. That is why this research recommends the quick first steps by the governmental and non-governmental bodies for promoting the possible outcomes of climate change impact. Perhaps the researchers believe that the most urgent priority for effective awareness of climate change and other initiatives to mitigate this problem the young citizens, both male and female can play an important role to engage with all sectors of society. As the role of media is very important in covering climate change information. Above all an honest and open dialogue about the risks of climate change in Malaysia between young representative and the policy makers is an essential first step to mitigate this problem in further.

## 6. Conclusion and Direction for Future Research

This study was undertaken to examine the brief review of climate change and its impact and perceived awareness of the young citizens in Malaysia. This study also explores that young segments in Malaysia feel about the climate change and perceived its intensity of physical and psychological aspects. There are few limitations exist in the research the first limitation was based on the sample area for the study, which is confined to the only Klang Valley area in Malaysia. Second, this research used convenient sampling procedure to collect the data. The outcome of this research will assists the policy makers to understand the overall perceived awareness of climate change by the younger citizens in Malaysia. The researchers believe that these handful ideas about this particle segment will assist them to introduce new initiatives to mitigate the impact of climate change. However, researchers still predict that further research efforts are being needed to examine the antecedents of awareness development under the mind of mass people in regards to climate change. Above all the researchers strongly agree that the best way to adjust the climate change is to involve all individuals in the society.
